# Risk factors and modes of failure in the modern dual mobility implant. A systematic review and meta-analysis

**DOI:** 10.1186/s12891-021-04404-4

**Published:** 2021-06-14

**Authors:** Fu-Yuan Pai, Hsuan-Hsiao Ma, Te-Feng Arthur Chou, Tsan-Wen Huang, Kuo-Chin Huang, Shang-Wen Tsai, Cheng-Fong Chen, Wei-Ming Chen

**Affiliations:** 1 Department of Orthopaedics and Traumatology, Taipei Veterans General Hospital, No. 201, Sec 2, Shi-Pai Road, Taipei 112, Taiwan; 2 Department of Orthopaedics, School of Medicine, National Yang-Ming Chiao-Tung University, Taipei, Taiwan; 3 Chang Gung University College of Medicine, Taoyuan, Taiwan; 4 Department of Orthopaedic Surgery, Chang-Gung Memorial Hospital, Chiayi, Taiwan

**Keywords:** Dislocation, Dual mobility, Implant failure, Instability, Outcome, Revision total hip arthroplasty, Risk factor, Total hip arthroplasty

## Abstract

**Background:**

The aims of this meta-analysis were to: (1) validate the outcome of modern dual mobility (DM) designs in patients who had undergone primary and revision total hip arthroplasty (THA) procedures and (2) to identify factors that affect the outcome.

**Methods:**

We searched for studies that assessed the outcome of modern DM-THA in primary and revision procedures that were conducted between January, 2000 to August, 2020 on PubMed, MEDLINE, Cochrane Reviews and Embase. The pooled incidence of the most common failure modes and patient reported outcomes were evaluated in patients who have received: (1) primary THA, (2) revision THA for all causes or (3) for recurrent dislocation. A meta-regression analysis was performed for each parameter to determine the association with the outcome. The study design of each study was assessed for potential bias and flaws by using the quality assessment tool for case series studies.

**Results:**

A total of 119 studies (N= 30016 DM-THAs) were included for analysis. The mean follow-up duration was 47.3 months. The overall implant failure rate was 4.2% (primary: 2.3%, revision for all causes: 5.5%, recurrent dislocation: 6.0%). The most common failure modes were aseptic loosening (primary: 0.9%, revision for all causes: 2.2%, recurrent dislocation: 2.4%), septic loosening (primary:0.8%, revision for all causes: 2.3%, recurrent dislocation: 2.5%), extra-articular dislocation (primary:0.6%, revision for all causes:1.3%, recurrent dislocation:2.5%), intra-prosthetic dislocation (primary:0.8%, revision for all causes:1.0%, recurrent dislocation:1.6%) and periprosthetic fracture (primary:0.9%, revision for all causes:0.9%, recurrent dislocation:1.3%). The multi-regression analysis identified younger age (β=-0.04, 95% CI -0.07 – -0.02) and female patients (β=3.34, 95% CI 0.91–5.78) were correlated with higher implant failure rate. Age, gender, posterolateral approach and body mass index (BMI) were not risk factors for extra-articular or intra-prosthetic dislocation in this cohort. The overall Harris hip score and Merle d’Aubign*é* score were 84.87 and 16.36, respectively. Level of evidence of this meta-analysis was IV.

**Conclusion:**

Modern dual-mobility designs provide satisfactory mid-term implant survival and clinical performance. Younger age and female patients might impact the outcome after DM-THA. Future research directions should focus on, (1) long-term outcome of modern dual-mobility design, including specific concerns such as intra-prosthetic dislocation and elevated metal ion, and (2) cost-effectiveness analysis of dual-mobility implant as an alternative to conventional THA for patients who are at high risk of dislocation.

**Supplementary Information:**

The online version contains supplementary material available at 10.1186/s12891-021-04404-4.

## Background

Prosthetic dislocation is one of the most common cause of implant failure after total hip arthroplasty (THA) [1]. The reported dislocation rate after primary THAs is 0.3-10% [2–4] and is much higher after revision THAs (5-30%) [5–7]. The cause of a dislocated prosthesis can be multifactorial, including both surgeon and patient related factors [8–18]. Several design changes have been made on the prosthesis to resolve this. Currently, dual mobility (DM) THA is one of the most successful designs to reduce the risk of dislocation [19]. The concept of DM was invented by Gilles Bousquet and Andrè Rambert in France in 1973 [19]. The design included Charnley’s low-friction principle and the theory of McKee and Watson-Farrar, which increased the femoral head-to-neck ratio, extending the “jumping” distance in order to prevent dislocations [20–23]. The first generation DM design was associated with higher aseptic loosening and intra-prosthetic dislocation (IPD) rate, which resulted from polyethylene wear, suboptimal fixation and surface coating of the acetabular component [24–30]. In the late 1990’s, a newer DM design was introduced with several modifications including modular design, shape, surface coating and highly cross-linked polyethylene to reduce the rate of aseptic loosening and IPD [31–34].

Compared with the fixed-bearing THA, several meta-analyses have validated a lower dislocation rate using DM articulation in both primary [35–37] and revision THA procedures [36–39]. Despite the established efficacy of DM articulation in preventing dislocation, it is with clinical importance to validate the overall implant survival and failure modes of this unique design. These studies could only provide results of inferential statistics rather than descriptive statistics with regard to the outcome after DM-THA because the included studies represented only a small number of DM-THA used in primary and revision THA procedures [36–39]. To our knowledge, the most recent and comprehensive systematic review discussing the outcome after DM-THA was conducted by Darrith et al. [40] The authors reviewed studies published from 2007 to 2016, including 54 studies with 14345 primary and revision THA procedures. They reported the overall failure rate (primary: 2.0%, revision: 3.4%) and incidence of common failure modes including aseptic loosening (primary: 1.3%, revision: 1.4%), extra-articular dislocation (primary: 0.46%, revision: 2.2%) and intra-prosthetic dislocation (primary: 1.1%, revision: 0.3%). However, this review included a mixture of the 1^st^ generation and modern (2^nd^ and 3^rd^ generations) DM designs. Several important modes of implant failure such as septic loosening and periprosthetic fracture were not analyzed in this review. Moreover, the number of articles regarding the outcome of modern DM-THA have doubled since 2016 [41–115]. Therefore, an up-to-date meta-analysis is essential to validate the outcome of modern DM-THA. Our primary objective was to identify the overall implant failure rate and several common failure modes including aseptic loosening, septic loosening, extra-articular dislocation, intra-prosthetic dislocation and periprosthetic fracture. The secondary objective was to determine risk factors predisposing to implant failure and the functional performance of these patients after surgery.

## Methods

**Fig. 1:**
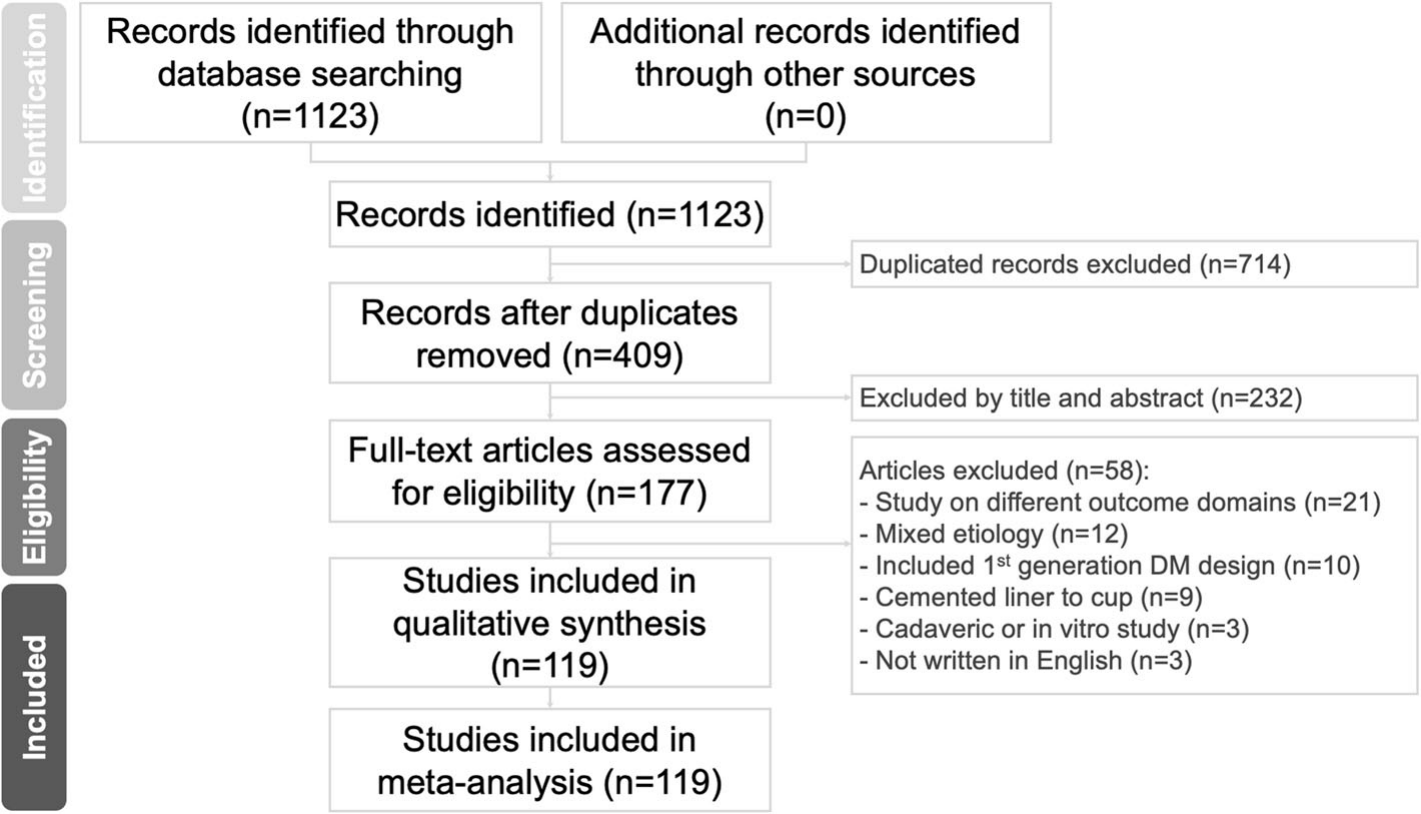
Preferred reporting items for systematic reviews and meta-analysis (PRISMA) flow diagram for the searching and identification of included studies

We completed a comprehensive search on PubMed, MEDLINE, Cochrane Reviews and Embase for studies that reported outcome in patients who had undergone dual mobility total hip arthroplasty (DM-THA) published from the earliest record to August, 2020. The search was completed in accordance to the Preferred Reporting Items for Systematic Reviews and Meta-analysis (PRISMA) statement. The following terms were used in variable combinations: total hip arthroplasty, total hip replacement and dual mobility. Two authors (FYP, SWT) independently searched and screened the titles and abstracts for relevant studies. If there was disagreement, a third author (HHM) was consulted for a consensus. The bibliographies of the included studies were manually reviewed for relevant references. The search strategy is shown in Fig. 1.

We included original articles written in English that validated the outcome in patients who had undergone DM-THA for all kinds of indications including primary THA, revision THA or recurrent dislocation. We excluded review articles, letter to the editor, expert opinion, biomechanical studies, articles not written in English, study period earlier than 2000 or studies in which data were not obtainable. The included studies must contain at least one of the primary (e.g. overall implant failure rate, failure modes including aseptic loosening, septic loosening, extra-articular dislocation, intra-prosthetic dislocation and periprosthetic fracture) or secondary outcome domains (e.g. functional scores). Two authors (FYP, SWT) examined all relevant studies and obtained data from the texts. If none of the above outcome domains can be obtained from the study, then we will exclude the study. For comparative studies (e.g. hemiarthroplasty or THA vs DM-THA), we extracted data from the DM-THA group if possible. If there was uncertainty regarding the data from the study, we contacted the authors for clarifications.

Two authors (FYP, SWT) examined all relevant studies and extracted data using a predetermined form. The primary aim was to determine the overall implant failure rate and failure modes including aseptic loosening, septic loosening, extra-articular dislocation, intra-prosthetic dislocation and periprosthetic fracture. We further validated these rates stratified by indications including primary THA, revision THA for all causes or for recurrent dislocation. The secondary aim was to identify risk factors for implant failures and to evaluate the functional outcome using Harris hip score [116] and Merle d’Aubign*é* score [117]. We recorded the first author, year, study design, number of THA procedures, indications, age, follow-up duration, implant brand and outcome parameters in Table 1.

**Table 1:** Characteristics of included studies

Author, Year	Study design	No. of THA procedure	Indications	Mean age (yrs)	Follow up duration (m)	Implant type	A	B	C	D	E	F	G	H
2020 Tabori-jensen	Prospective series	59	Primary	75	24	1	V	V	V	V	V	V	V	
2020 Schmidt	Retrospective series	184	Revision	69	24	2, 3	V	V	V	V	V	V		
2020 Rashed	Prospective series	31	Primary	66.4	12	4			V	V			V	
2020 Nessler	Retrospective series	93	Primary	65.5	32.4	5	V	V	V	V	V			
2020 Laende	Retrospective series	27	Primary	63	36	6	V	V	V	V	V	V		
2020 Klemt	Retrospective series	42	Revision	55	48	1, 5, 6, 10, 13	V	V	V	V	V	V		
2020 Hoggett	Retrospective series	28	Recurrent dislocation	80	55	3, 7	V	V	V	V	V	V		
2020 Favreau	Retrospective series	40	Revision	77	54	3	V	V	V	V	V	V	V	V
2020 Dubin	Retrospective series	664	Primary	61.7	25	5, 6	V	V	V	V	V	V	V	
2020 Dubin (Arthroplasty Today)	Retrospective series	142	Primary	67	68.4	6	V	V	V	V	V	V		
2020 de l’Escalopier	Retrospective series	84	Revision	71	65.3	8, 9	V	V	V	V	V	V		V
2020 Colacchio	Retrospective series	29	Revision	61.4	47	6, 10	V	V	V	V	V	V		
2020 Civinini	Retrospective series	37	Revision	63.7	61.2	5							V	
2020 Ait Mokhtar	Retrospective series	148	Primary	78	38	2	V	V	V	V	V	V		
2020 Abdel	Retrospective series	126	Revision	66	43.2	5	V	V	V	V	V	V		
2019 Ukaj	Prospective series	47	Primary	78.1	36	2	V	V	V	V	V	V	V	
2019 Tabori-jensen, Arch	Retrospective series	997	Primary	80.5	64.8	1, 11	V	V	V	V	V	V		
2019 Schmidt-braekling	Retrospective series	77	Revision	68.5	63.6	1, 4	V	V	V	V	V	V	V	
2019 Nonne	Retrospective series	60	Primary	87.6	28.3	12	V	V	V	V	V	V	V	
2019 Neil Wheelton	Retrospective series	54	Revision	78	22.8	NR	V	V	V	V	V	V		
2019 Nam	Prospective series	43	Primary	52.6	24	5							V	
2019 Markel	Prospective series	21	Primary	61.7	24	5							V	
2019 Li	Retrospective series	94	Revision	63.6	37.8	5			V	V		V		
2019 Kreipke	Retrospective series	2277	Primary	75.5	35.9	1, 11, 13	V	V	V	V	V	V		
2019 Jones	Retrospective series	151	Primary	82	43.2	6	V	V	V	V	V	V		
2019 Jobory	Retrospective series	4520	Primary	77	25.2	1, 11, 13	V	V			V	V		
2019 Iorio	Retrospective series	30	Primary	82	12	2	V	V	V	V	V	V		
2019 Huang	Retrospective series	315	Revision	65.8	39.6	5	V	V	V	V	V	V	V	
2019 Huang	Retrospective series	107	Recurrent dislocation	65.8	39.6	5			V	V				
2019 Gaillard	Retrospective series	138	Primary	68	152.4	11	V	V	V	V	V	V	V	V
2019 Fessy	Retrospective series	541	Primary	73.6	103.2	3	V	V	V	V	V	V	V	
2019 Fahad	Retrospective series	27	Primary	69.3	19	NR			V	V	V		V	
2019 Dubin	Retrospective series	287	Primary	67.8	34.3	6	V	V	V	V	V	V	V	
2019 Dubin	Retrospective series	287	Primary	67.9	34.3	5	V	V	V	V	V	V	V	
2019 Dikmen	Prospective series	34	Revision	66.1	42.24	13	V	V	V	V	V	V	V	
2019 Cypres	Retrospective series	244	Primary	63.8	142.8	13	V	V	V	V	V	V		V
2019 Chalmers	Retrospective series	24	Revision	63	48	5							V	
2019 Canton	Retrospective series	31	Primary	76.7	67.2	1	V	V	V	V			V	
2019 Boulat	Retrospective series	33	Primary	74	44	3	V	V	V	V	V	V	V	V
2019 Bloemheuvel	Retrospective series	3038	Primary	70	36	1, 2, 11, 13, 14	V	V	V	V	V	V		
2019 Bloemheuvel	Retrospective series	4637	Revision	74	72	1, 2, 11, 13, 14	V	V			V	V		
2019 Assi(J Arthroplasty)	Retrospective series	125	Primary	78.1	61.2	1, 2	V	V	V	V			V	
2019 Assi(Int Orthop)	Retrospective series	16	Revision	69.2	72.9	NR	V	V	V	V	V	V	V	
2019 Assi(Hip Int.)	Retrospective series	229	Primary	62	70	1, 2	V	V	V	V	V	V	V	
2019 Addona	Retrospective series	107	Primary	NR	NR	5; 15			V	V		V		
2019 Addona	Retrospective series	47	Revision	NR	NR	5; 15			V	V		V		
2018 Tabori-Jensen	Retrospective series	124	Primary	74.7	33.6	11	V	V	V	V	V	V	V	
2018 Stucinskas	Retrospective series	247	Revision	72	24	1; 2	V	V	V	V	V	V		
2018 Spaans	Retrospective series	102	Recurrent dislocation	73.1	27.6	1	V	V	V	V	V	V		
2018 Rashed	Prospective series	32	Primary	66.4	12	4	V	V	V	V	V	V	V	
2018 Perrin	Prospective series	24	Revision	79.5	6	NR	V	V	V	V	V	V		
2018 Ozden	Retrospective series	20	Revision	64.5	38.1	13	V	V	V	V	V	V	V	
2018 Marie-hardy	Retrospective series	16	Primary	69.6	29	3	V	V	V	V	V	V		V
2018 Lange	Retrospective series	40	Recurrent dislocation	64	36	5; 6	V	V	V	V	V	V		
2018 Kim	Retrospective series	84	Primary	73.1	21.7	5			V	V			V	
2018 Kavcic	Retrospective series	173	Primary	76.8	92.4	1	V	V	V	V	V	V	V	
2018 Kasparek	Retrospective series	11	Revision	64	31	5; 6	V	V	V	V	V	V	V	
2018 Hwang	Prospective series	167	Primary	72	22	10	V	V	V	V	V	V	V	
2018 Harwin	Retrospective series	85	Revision	67	48	5	V	V	V	V	V	V	V	
2018 Hartzler	Retrospective series	126	Revision	66	40	5			V	V		V		
2018 Diamond	Retrospective series	60	Revision	65.5	38.6	5	V	V	V	V	V	V		
2018 Chalmers	Retrospective series	14	Recurrent dislocation	65	37	5	V	V	V	V	V	V	V	
2018 Boukebous	Retrospective series	98	Primary	77.8	25.9	16, 17			V	V				
2018 Assi	Retrospective series	30	Primary	54.9	51	1; 2; 19	V	V	V	V	V	V	V	
2017 Viste	Retrospective series	334	Revision	NR	84	3	V	V	V	V	V	V		
2017 Tarasevicius	Retrospective series	620	Revision	63.2	30	1; 2	V	V	V	V	V	V		
2017 Sutter	Retrospective series	64	Revision	59	38	5	V	V	V	V	V	V		
2017 Rowan	Retrospective series	136	Primary	48.5	38.4	5; 6	V	V	V	V	V	V	V	
2017 Puch	Prospective series	103	Primary	49.9	132	20	V	V	V	V	V	V	V	V
2017 Puch	Prospective series	217	Primary	72.3	149	20	V	V	V	V	V	V	V	V
2017 Ochi	Retrospective series	33	Primary	80	15.8	5	V	V	V	V	V	V		
2017 Nam	Prospective series	26	Primary	52.8	12	5							V	
2017 Martz	Retrospective series	25	Primary	44	129.8	3	V	V	V	V	V	V	V	V
2017 Lebeau	Retrospective series	62	Revision	75.5	77	2 (1st-gen)	V	V	V	V	V	V	V	V
2017 Hernigou	Retrospective series	35	Revision	73	84	2, 21	V	V	V	V	V	V		V
2017 Henawy	Prospective series	24	Primary	68	12	3	V	V	V	V	V	V	V	V
2017 Hamadouche	Retrospective series	51	Revision	71.4	60	8	V	V	V	V	V	V		V
2017 Graversen	Retrospective series	20	Primary	83	12	1	V	V	V	V	V	V		
2017 Gonzalez	Prospective series	150	Revision	73	6	13; 22	V	V	V	V	V	V		
2017 Ferreira	Retrospective series	553	Primary	71.2	36	2	V	V	V	V	V	V		
2017 Ferreira	Retrospective series	83	Primary	81.7	36	2	V	V	V	V	V	V		
2017 Epinette	Retrospective series	321	Primary	48.1	32.4	5, 6	V	V	V	V	V	V	V	
2017 Chalmers	Retrospective series	16	Revision	75	36	5	V	V	V	V	V	V	V	
2017 Batailler	Retrospective series	302	Primary	73	14	2, 23	V	V	V	V	V	V	V	V
2016 Nich	Retrospective series	45	Primary	86.7	23.8	6; 24	V	V	V	V	V	V		V
2016 Morin	Retrospective series	40	Primary	19.2	60	NR	V	V	V	V	V	V		
2016 Jauregui	Retrospective series	60	Revision	57	30	5	V	V	V	V	V	V	V	
2016 Homma	Retrospective series	60	Primary	75.6	6	5; 6	V	V	V	V	V	V		
2016 Haughom	Retrospective series	24	Primary	50.2	3	NR			V	V				
2016 Griffin	Prospective series	10	Primary	>60	12	3	V	V	V	V	V	V		
2016 Chughtai	Retrospective series	410	Primary	64	36	5	V	V	V	V	V	V	V	
2016 Carulli	Retrospective series	31	Recurrent dislocation	75.4	45.6	1	V	V	V	V	V	V	V	
2015 Wegrzyn	Retrospective series	994	Revision	70	87.6	11	V	V	V	V				
2015 Vigdorchik	Retrospective series	485	Primary	66	24	6	V	V	V	V	V	V	V	
2015 Vermersch	Prospective series	86	Primary	72	27	3	V	V	V	V	V	V	V	V
2015 van Heumen	Retrospective series	50	Recurrent dislocation	67	29	1	V	V	V	V	V	V		
2015 Snir	Retrospective series	18	Revision	50.6	26.5	5; 6; 10	V	V	V	V	V	V	V	
2015 Simian	Retrospective series	74	Revision	67.9	87.6	17; 18; 25	V	V	V	V	V	V	V	V
2015 Mohammed	Retrospective series	20	Primary	70.8	22	NR	V	V	V	V	V	V		
2015 Mohammed	Retrospective series	24	Revision	76.4	22	NR	V	V	V	V	V	V		
2015 Epinette	Prospective series	143	Primary	70.6	50	6	V	V	V	V	V	V	V	V
2015 Bel	Retrospective series	18	Primary	84	36	3	V	V	V	V	V	V		
2014 Wegrzyn	Prospective series	61	Revision	67	86	11	V	V	V	V	V	V	V	
2014 Prudhon	Prospective series	79	Revision	62.5	24	7	V	V	V	V	V	V		
2014 Jakobsen	Retrospective series	56	Recurrent dislocation	72	44	11	V	V	V	V	V	V	V	
2014 Epinette	Prospective series	437	Primary	74.2	24	6	V	V	V	V	V	V	V	V
2014 Caton	Retrospective series	105	Primary	78	120	2	V	V	V	V	V	V		
2014 Bensen	Retrospective series	175	Primary	75.2	21.7	11	V	V	V	V	V	V		
2013 Tarasevicius	Retrospective series	41	Primary	75	12	1	V	V	V	V	V	V		
2013 Saragaglia	Retrospective series	29	Recurrent dislocation	75.6	46	1; 3; 20; 24	V	V	V	V	V	V		V
2013 Sanders	Retrospective series	10	Primary	54	39	1	V	V	V	V	V	V		
2013 Prudhon	Retrospective series	105	Primary	78	91	2	V	V	V	V	V	V		V
2012 Vasukutty	Retrospective series	143	Revision	77	42	NR	V	V	V	V	V	V		
2012 Pattyn	Retrospective series	36	Revision	70	16	26	V	V	V	V	V	V		
2012 Hamadouche	Retrospective series	119	Primary	67	72	9	V	V	V	V				V
2012 Hailer	Retrospective series	228	Recurrent dislocation	75	24	1	V	V	V	V	V	V		
2012 Civinini	Prospective series	33	Revision	69	36	1	V	V	V	V	V	V	V	
2012 Adam	Prospective series	214	Primary	83	9	NR	V	V	V	V	V	V		
2011 Schneider	Retrospective series	96	Revision	69.9	41	3	V	V	V	V	V	V		V
2011 Bouchet	Retrospective series	105	Primary	76.6	28	1; 3; 20; 24			V	V		V		
2010 Tarasevicius	Retrospective series	42	Primary	75	12	1			V	V		V		
2010 Hamadouche	Retrospective series	47	Recurrent dislocation	71.3	51.4	8	V	V	V	V	V	V		V
2009 Guyen	Retrospective series	54	Recurrent dislocation	66.5	48	11	V	V	V	V	V	V	V	
2008 Langlais	Retrospective series	88	Revision	72	36	8	V	V	V	V	V	V		V
2008 Bauchu	Retrospective series	121	Primary	69	74.4	13	V	V	V	V	V	V		V

A: aseptic loosening; B; septic loosening or PJI; C: extra-dislocation; D: Intra-dislocation; E: Periprosthetic fracture; F: implant failure; G; HHS; H: Merle D’Aubigne scores

1: Avantage (Zimmer Biomet, Warsaw, Indiana, USA); 2: Quattro (Groupe Lépine, Genay, France ); 3. Novae cup or Novae Sunfit cup (Serf, Décines, France); 4. EcoFit 2M cup (Ecofit, implantcast, Buxtehude, Germany); 5. Stryker MDM (Stryker, Mahwah, New Jersey, USA); 6. Stryker ADM (Stryker, Mahwah, New Jersey, USA); 7. ADES (Zimmer Biomet, Warsaw, Indiana, USA); 8. Medial cup (Aston Medical, Saint-Étienne, France); 9. Tregor cup (Aston Medical, Saint-Étienne, France); 10. Biomet Active Articulation E1 (Biomet Orthopedics, Warsaw, Indiana, USA); 11. Saturne (Amplitude, Valence, France); 12. Dualis acetabular cup (Gruppo Bioimpianti, Peschiera Borromeo, Milano, Italy); 13. Polarcup (Smith & Nephew AG, Aarau, Switzerland); 14. SeleXys DS cup (Mathys European Orthopaedics, Bettlach, Switzerland); 15. G7 DM (Zimmer Biomet, Warsaw, Indiana, USA); 16. Galiléa (SEM, Créteil, France); 17. Evora (SEM, Créteil, France); 18. DMS (SEM, Créteil, France); 19. Hip’n Go dual mobility (FH orthopedics, Mulhouse, France); 20. Gyros cup (Depuy, Warsaw, IN, USA); 21. Ceraver DM device (Ceraver Osteal, Roissy, France); 22. Versafit DM cup (Medacata international, Castel San Pietro, Switzerland); 23. Tornier DM cup (Tornier, Montbonnot-Saint-Martin, France); 24. Stafit (Zimmer, Etupes, France); 25. Mobilite (Tornier, Montbonnot-Saint-Martin, France); 26. Apogee DM socket (Biotechni Inc., Marseille, France)

Two authors (FYP, SWT) independently evaluated the methodological quality of the included studies using the NIH Quality Assessment Tool for Case Series Studies and Case Control Studies [118, 119]. To assess the quality of case series study, the highest score on this scale is 9. A score between 7 and 9, 4 and 6, less than 4 were defined as “good”, “fair” and “poor”, respectively. For the quality of case control study, the highest score on this scale is 12. A score between 8 and 12, 5 and 7, less than 5 were defined as “good”, “fair” and “poor”, respectively. If there were disagreement, we consulted a third author (HHM). (Tables 2 and 3) Of the 119 included studies, the methodological quality was considered “good” in 72 (60.5%) studies and “fair” in 47 (39.5%) studies.

**Table 2:** Study assessment based on quality assessment tool for case series studies

Criteria	2020 Nessler et al.	2020 Laende et al.	2020 Favreau et al.	2020 Dubin (Arthroplasty Today) et al.	2020 de l’Escalopier et al.	2020 Colacchio et al.	2020 Civinini et al.	2020 Ait Mokhtar et al	2019 Tabori-jensen et al.			
1. Was the study question or objective clearly stated?	Y	Y	Y	Y	Y	Y	Y	Y	Y			
2. Was the study population clearly and fully described, including a case definition?	Y	Y	Y	Y	Y	Y	Y	Y	Y			
3. Were the cases consecutive?	N	Y	N	N	Y	N	N	Y	Y			
4. Were the subjects comparable?	N	N	N	N	N	N	N	N	N			
5. Was the intervention clearly described?	Y	Y	Y	Y	Y	Y	Y	Y	Y			
6. Were the outcome measures clearly defined, valid, reliable and implemented consistently across all study participants?	Y	Y	Y	Y	Y	Y	Y	Y	Y			
7. Was the length of follow-up adequate?	Y	Y	Y	Y	Y	Y	Y	Y	Y			
8. Were the statistical methods well-described?	Y	Y	Y	Y	Y	Y	Y	N	Y			
9. Were the results well-described?	Y	Y	Y	Y	Y	Y	Y	Y	Y			
Quality of the cohort study (score)	7	8	7	7	8	7	7	7	8			
Criteria	2019 Schmidt-braekling et al.	2019 Neil Wheelton et al.	2019 Nam et al.	2019 Markel et al.	2019 Jones et al.	2019 Huang et al.	2019 Gaillard et al.	2019 Fessy et al.	2019 Dikmen et al.	2019 Cypres et al.	2019 Chalmers et al.	
1. Was the study question or objective clearly stated?	Y	Y	Y	Y	Y	Y	Y	Y	Y	Y	Y	
2. Was the study population clearly and fully described, including a case definition?	Y	Y	Y	Y	Y	Y	Y	Y	Y	Y	Y	
3. Were the cases consecutive?	Y	Y	N	N	Y	Y	Y	Y	Y	N	N	
4. Were the subjects comparable?	N	N	N	N	N	N	N	N	N	N	N	
5. Was the intervention clearly described?	Y	Y	Y	Y	Y	Y	Y	Y	Y	Y	Y	
6. Were the outcome measures clearly defined, valid, reliable and implemented consistently across all study participants?	Y	Y	Y	Y	Y	Y	Y	Y	Y	Y	Y	
7. Was the length of follow-up adequate?	Y	N	Y	Y	Y	Y	Y	Y	Y	Y	Y	
8. Were the statistical methods well-described?	Y	N	Y	Y	N	Y	Y	Y	Y	Y	Y	
9. Were the results well-described?	Y	Y	Y	Y	Y	Y	Y	Y	Y	Y	Y	
Quality of the cohort study (score)	8	6	7	7	7	8	8	8	8	7	7	
Criteria	2019 Canton et al.	2019 Boulat et al.	2019 Assi (J Arthroplasty) et al.	2019 Assi (Hip Int.) et al.	2019 Addona et al.	2018 Rashed et al.	2018 Ozden et al.	2018 Marie-hardy et al.	2018 Lange et al.	2018 Kavcicet al.	2018 Kasparek et al.	
1. Was the study question or objective clearly stated?	Y	Y	Y	Y	Y	Y	Y	Y	Y	Y	Y	
2. Was the study population clearly and fully described, including a case definition?	Y	Y	Y	Y	Y	Y	Y	Y	Y	Y	Y	
3. Were the cases consecutive?	N	N	N	N	Y	N	Y	Y	N	Y	N	
4. Were the subjects comparable?	N	N	N	N	N	N	N	N	N	N	N	
5. Was the intervention clearly described?	Y	Y	Y	Y	Y	Y	Y	Y	Y	Y	Y	
6. Were the outcome measures clearly defined, valid, reliable and implemented consistently across all study participants?	Y	Y	Y	Y	Y	Y	Y	Y	Y	Y	Y	
7. Was the length of follow-up adequate?	Y	Y	Y	Y	N	N	Y	Y	Y	Y	Y	
8. Were the statistical methods well-described?	Y	Y	Y	Y	N	Y	Y	N	Y	Y	Y	
9. Were the results well-described?	Y	Y	Y	Y	Y	Y	Y	Y	Y	Y	Y	
Quality of the cohort study (score)	7	7	7	7	6	6	8	7	7	8	7	
Criteria	2018 Hwang et al.	2018 Diamond et al.	2018 Chalmers et al.	2018 Assi et al.	2017 Viste et al.	2017 Sutter et al.	2017 Puch et al.	2017 Nam et al.	2017 Martz et al.	2017 Lebeau et al.	2017 Henawy et al.	2017 Hamadouche et al.
1. Was the study question or objective clearly stated?	Y	Y	Y	Y	Y	Y	Y	Y	Y	Y	Y	Y
2. Was the study population clearly and fully described, including a case definition?	Y	Y	Y	Y	Y	Y	Y	Y	Y	Y	Y	Y
3. Were the cases consecutive?	N	Y	N	Y	Y	Y	Y	N	N	N	Y	N
4. Were the subjects comparable?	N	N	N	N	N	N	N	N	N	N	N	N
5. Was the intervention clearly described?	Y	Y	Y	Y	Y	Y	Y	Y	Y	Y	Y	Y
6. Were the outcome measures clearly defined, valid, reliable and implemented consistently across all study participants?	Y	Y	Y	Y	Y	Y	Y	Y	Y	Y	Y	Y
7. Was the length of follow-up adequate?	N	Y	Y	Y	Y	Y	Y	N	Y	Y	N	Y
8. Were the statistical methods well-described?	Y	Y	N	N	Y	Y	Y	Y	Y	Y	N	Y
9. Were the results well-described?	Y	Y	Y	Y	Y	Y	Y	Y	Y	Y	Y	Y
Quality of the cohort study (score)	6	8	6	7	8	8	8	6	7	7	6	7
Criteria	2017 Graversen et al.	2017 Ferreira et al.	2017 Epinette et al.	2016 Nich et al.	2016 Morin et al.	2016 Chughtai et al.	2016 Carulli et al.	2015 Wegrzyn et al.	2015 Vigdorchik et al.	2015 Vermersch et al.	2015 van Heumen et al.	
1. Was the study question or objective clearly stated?	Y	Y	Y	Y	Y	Y	Y	Y	Y	Y	Y	
2. Was the study population clearly and fully described, including a case definition?	Y	Y	Y	Y	Y	Y	Y	Y	Y	Y	Y	
3. Were the cases consecutive?	N	N	N	N	N	N	N	N	N	Y	Y	
4. Were the subjects comparable?	N	N	N	N	N	N	N	N	N	N	N	
5. Was the intervention clearly described?	Y	Y	Y	Y	Y	Y	Y	Y	Y	Y	Y	
6. Were the outcome measures clearly defined, valid, reliable and implemented consistently across all study participants?	Y	Y	Y	Y	Y	Y	Y	Y	Y	Y	Y	
7. Was the length of follow-up adequate?	N	Y	Y	Y	Y	Y	Y	Y	Y	Y	Y	
8. Were the statistical methods well-described?	Y	Y	Y	Y	Y	Y	N	N	Y	Y	Y	
9. Were the results well-described?	Y	Y	Y	Y	Y	Y	Y	Y	Y	Y	Y	
Quality of the cohort study (score)	6	7	7	7	7	7	6	6	7	8	8	
Criteria	2015 Snir et al.	2015 Simian et al.	2015 Mohammed et al.	2014 Wegrzyn et al.	2014 Prudhon et al.	2014 Jakobsen et al.	2013 Saragaglia et al.	2013 Sanders et al.	2013 Prudhon et al.	2012 Vasukutty et al.	2012 Pattyn et al.	
1. Was the study question or objective clearly stated?	Y	Y	Y	Y	Y	Y	Y	Y	Y	Y	Y	
2. Was the study population clearly and fully described, including a case definition?	Y	Y	Y	Y	Y	Y	Y	Y	Y	Y	Y	
3. Were the cases consecutive?	Y	N	N	N	Y	Y	N	Y	Y	Y	N	
4. Were the subjects comparable?	N	N	N	N	N	N	N	N	N	N	N	
5. Was the intervention clearly described?	Y	Y	Y	Y	Y	Y	Y	Y	Y	Y	Y	
6. Were the outcome measures clearly defined, valid, reliable and implemented consistently across all study participants?	Y	Y	Y	Y	Y	Y	Y	Y	Y	Y	Y	
7. Was the length of follow-up adequate?	N	Y	N	Y	Y	Y	Y	Y	Y	Y	N	
8. Were the statistical methods well-described?	N	Y	N	Y	Y	Y	N	Y	Y	Y	N	
9. Were the results well-described?	Y	Y	Y	Y	Y	Y	Y	Y	Y	Y	Y	
Quality of the cohort study (score)	6	7	5	7	8	8	6	8	8	8	5	
Criteria	2012 Hamadouche et al.	2012 Hailer et al.	2012 Civinini et al.	2012 Adam et al.	2011 Schneider et al.	2010 Hamadouche et al.	2009 Guyen et al.	2008 Langlais et al.	2008 Bauchu et al.			
1. Was the study question or objective clearly stated?	Y	Y	Y	Y	Y	Y	Y	Y	Y			
2. Was the study population clearly and fully described, including a case definition?	Y	Y	Y	Y	Y	Y	Y	Y	Y			
3. Were the cases consecutive?	N	N	N	N	N	N	N	N	Y			
4. Were the subjects comparable?	N	N	N	N	N	N	N	N	N			
5. Was the intervention clearly described?	Y	Y	Y	Y	Y	Y	Y	Y	Y			
6. Were the outcome measures clearly defined, valid, reliable and implemented consistently across all study participants?	Y	Y	Y	Y	Y	Y	Y	Y	Y			
7. Was the length of follow-up adequate?	Y	Y	Y	N	Y	Y	Y	Y	Y			
8. Were the statistical methods well-described?	Y	Y	Y	Y	Y	Y	Y	Y	N			
9. Were the results well-described?	Y	Y	Y	Y	Y	Y	Y	Y	Y			
Quality of the cohort study (score)	7	7	7	6	7	7	7	7	7			

Y= Yes, N= No; The maximum possible score on this scale is 9. “Good” was defined as a total score of 7-9; “fair” as a score 4-6, and “poor” as a score of less than 4.

**Table 3:** Study assessment based on quality assessment tool for case control studies

Criteria	2020 Tabori-jensen et al.	2020 Schmidt et al.	2020 Rashed et al.	2020 Klemt et al	2020 Hoggett et al.	2020 Dubin et al.	2020 Abdel et al	
1. Was the research question or objective in this paper clearly stated and appropriate?	Y	Y	Y	Y	Y	Y	Y	
2. Was the study population clearly specified and defined?	Y	Y	Y	Y	Y	Y	Y	
3. Did the authors include a sample size justification?	Y	N	Y	Y	N	N	N	
4. Were controls selected or recruited from the same or similar population that gave rise to the cases (including the same timeframe)?	Y	Y	Y	N	Y	Y	Y	
5. Were the definitions, inclusion and exclusion criteria, algorithms or processes used to identify or select cases and controls valid, reliable, and implemented consistently across all study participants?	Y	Y	Y	Y	Y	Y	Y	
6. Were the cases clearly defined and differentiated from controls?	Y	Y	Y	Y	Y	Y	Y	
7. If less than 100 percent of eligible cases and/or controls were selected for the study, were the cases and/or controls randomly selected from those eligible?	Y	N	Y	N	N	N	N	
8. Was there use of concurrent controls?	NR	NR	NR	NR	NR	NR	NR	
9. Were the investigators able to confirm that the exposure/risk occurred prior to the development of the condition or event that defined a participant as a case?	Y	Y	Y	Y	Y	Y	Y	
10. Were the measures of exposure/risk clearly defined, valid, reliable, and implemented consistently (including the same time period) across all study participants?	Y	Y	Y	N	N	Y	Y	
11. Were the assessors of exposure/risk blinded to the case or control status of participants?	Y	N	N	N	N	N	N	
12. Were key potential confounding variables measured and adjusted statistically in the analyses? If matching was used, did the investigators account for matching during study analysis?	N	N	N	N	N	N	N	
Quality of the cohort study (score)	10	7	9	6	6	7	7	
Criteria	2019 Ukaj et al.	2019 Nonne et al.	2019 Li et al.	2019 Kreipke et al.	2019 Jobory et al.	2019 Iorio et al.	2019 Fahad et al.	2019 Dubin et al.
1. Was the research question or objective in this paper clearly stated and appropriate?	Y	Y	Y	Y	Y	Y	Y	Y
2. Was the study population clearly specified and defined?	Y	Y	Y	Y	Y	Y	Y	Y
3. Did the authors include a sample size justification?	Y	N	Y	N	N	N	N	N
4. Were controls selected or recruited from the same or similar population that gave rise to the cases (including the same timeframe)?	Y	Y	Y	N	N	Y	Y	Y
5. Were the definitions, inclusion and exclusion criteria, algorithms or processes used to identify or select cases and controls valid, reliable, and implemented consistently across all study participants?	Y	Y	Y	Y	Y	Y	Y	Y
6. Were the cases clearly defined and differentiated from controls?	Y	Y	Y	Y	Y	Y	Y	Y
7. If less than 100 percent of eligible cases and/or controls were selected for the study, were the cases and/or controls randomly selected from those eligible?	Y	N	N	N	N	Y	N	N
8. Was there use of concurrent controls?	NR	NR	NR	NR	NR	NR	NR	NR
9. Were the investigators able to confirm that the exposure/risk occurred prior to the development of the condition or event that defined a participant as a case?	Y	Y	Y	Y	Y	Y	Y	Y
10. Were the measures of exposure/risk clearly defined, valid, reliable, and implemented consistently (including the same time period) across all study participants?	Y	Y	Y	N	N	Y	Y	Y
11. Were the assessors of exposure/risk blinded to the case or control status of participants?	Y	N	N	N	N	N	N	N
12. Were key potential confounding variables measured and adjusted statistically in the analyses? If matching was used, did the investigators account for matching during study analysis?	N	N	N	N	N	N	N	N
Quality of the cohort study (score)	10	7	8	5	5	8	7	7
Criteria	2019 Bloemheuvel, van Steenbergen et al.	2019 Bloemheuvel, Steenbergen et al.	2019 Assi (Int Orthop) et al.	2018 Tabori-Jensen et al.	2018 Stucinskas et al.	2018 Spaans et al.	2018 Perrin et al.	2018 Kim et al.
1. Was the research question or objective in this paper clearly stated and appropriate?	Y	Y	Y	Y	Y	Y	Y	Y
2. Was the study population clearly specified and defined?	Y	Y	Y	Y	Y	Y	Y	Y
3. Did the authors include a sample size justification?	N	N	N	N	N	N	Y	N
4. Were controls selected or recruited from the same or similar population that gave rise to the cases (including the same timeframe)?	N	N	Y	Y	Y	Y	Y	Y
5. Were the definitions, inclusion and exclusion criteria, algorithms or processes used to identify or select cases and controls valid, reliable, and implemented consistently across all study participants?	Y	Y	Y	Y	Y	Y	Y	Y
6. Were the cases clearly defined and differentiated from controls?	Y	Y	Y	Y	Y	Y	Y	Y
7. If less than 100 percent of eligible cases and/or controls were selected for the study, were the cases and/or controls randomly selected from those eligible?	N	N	N	N	N	N	N	N
8. Was there use of concurrent controls?	NR	NR	NR	NR	NR	NR	NR	NR
9. Were the investigators able to confirm that the exposure/risk occurred prior to the development of the condition or event that defined a participant as a case?	Y	Y	Y	Y	Y	Y	Y	Y
10. Were the measures of exposure/risk clearly defined, valid, reliable, and implemented consistently (including the same time period) across all study participants?	N	N	Y	Y	Y	Y	Y	Y
11. Were the assessors of exposure/risk blinded to the case or control status of participants?	N	N	N	N	N	N	N	N
12. Were key potential confounding variables measured and adjusted statistically in the analyses? If matching was used, did the investigators account for matching during study analysis?	N	N	N	N	N	N	N	N
Quality of the cohort study (score)	5	5	7	7	7	7	8	7
Criteria	2018 Harwin et al.	2018 Hartzler et al.	2018 Boukebous et al.	2017 Tarasevicius et al.	2017 Rowan et al.	2017 Ochi et al.	2017 Hernigou et al.	2017 Gonzalez et al.
1. Was the research question or objective in this paper clearly stated and appropriate?	Y	Y	Y	Y	Y	Y	Y	Y
2. Was the study population clearly specified and defined?	Y	Y	Y	Y	Y	Y	Y	Y
3. Did the authors include a sample size justification?	N	N	Y	N	N	N	N	N
4. Were controls selected or recruited from the same or similar population that gave rise to the cases (including the same timeframe)?	Y	Y	Y	N	Y	Y	Y	Y
5. Were the definitions, inclusion and exclusion criteria, algorithms or processes used to identify or select cases and controls valid, reliable, and implemented consistently across all study participants?	Y	Y	Y	Y	Y	Y	Y	Y
6. Were the cases clearly defined and differentiated from controls?	Y	Y	Y	Y	Y	Y	Y	Y
7. If less than 100 percent of eligible cases and/or controls were selected for the study, were the cases and/or controls randomly selected from those eligible?	N	N	N	N	N	Y	N	N
8. Was there use of concurrent controls?	NR	NR	NR	NR	NR	NR	NR	NR
9. Were the investigators able to confirm that the exposure/risk occurred prior to the development of the condition or event that defined a participant as a case?	Y	Y	Y	Y	Y	Y	Y	Y
10. Were the measures of exposure/risk clearly defined, valid, reliable, and implemented consistently (including the same time period) across all study participants?	Y	Y	Y	N	Y	Y	Y	Y
11. Were the assessors of exposure/risk blinded to the case or control status of participants?	N	N	N	N	N	N	N	N
12. Were key potential confounding variables measured and adjusted statistically in the analyses? If matching was used, did the investigators account for matching during study analysis?	N	N	N	N	N	N	N	N
Quality of the cohort study (score)	7	7	8	6	7	8	7	7
Criteria	2017 Chalmers et al.	2017 Batailler et al.	2016 Jauregui et al.	2016 Homma et al.	2016 Haughom et al.	2016 Griffin et al.	2015 Epinette et al.	2015 Bel et al.
1. Was the research question or objective in this paper clearly stated and appropriate?	Y	Y	Y	Y	Y	Y	Y	Y
2. Was the study population clearly specified and defined?	Y	Y	Y	Y	Y	Y	Y	Y
3. Did the authors include a sample size justification?	N	N	N	N	Y	N	N	N
4. Were controls selected or recruited from the same or similar population that gave rise to the cases (including the same timeframe)?	Y	Y	Y	Y	Y	Y	Y	Y
5. Were the definitions, inclusion and exclusion criteria, algorithms or processes used to identify or select cases and controls valid, reliable, and implemented consistently across all study participants?	Y	Y	Y	Y	Y	Y	Y	Y
6. Were the cases clearly defined and differentiated from controls?	Y	Y	Y	Y	Y	Y	Y	Y
7. If less than 100 percent of eligible cases and/or controls were selected for the study, were the cases and/or controls randomly selected from those eligible?	N	N	N	N	N	Y	N	Y
8. Was there use of concurrent controls?	NR	NR	NR	NR	NR	NR	NR	NR
9. Were the investigators able to confirm that the exposure/risk occurred prior to the development of the condition or event that defined a participant as a case?	Y	Y	Y	Y	Y	Y	Y	Y
10. Were the measures of exposure/risk clearly defined, valid, reliable, and implemented consistently (including the same time period) across all study participants?	Y	Y	Y	Y	Y	Y	Y	Y
11. Were the assessors of exposure/risk blinded to the case or control status of participants?	N	N	N	N	N	Y	N	N
12. Were key potential confounding variables measured and adjusted statistically in the analyses? If matching was used, did the investigators account for matching during study analysis?	N	Y	N	N	N	N	N	N
Quality of the cohort study (score)	7	8	7	7	8	9	7	8
Criteria	2014 Epinette et al.	2014 Caton et al.	2014 Bensen et al.	2013 Tarasevicius et al.	2011 Bouchet et al.	2010 Tarasevicius et al.		
1. Was the research question or objective in this paper clearly stated and appropriate?	Y	Y	Y	Y	Y	Y		
2. Was the study population clearly specified and defined?	Y	Y	Y	Y	Y	Y		
3. Did the authors include a sample size justification?	N	N	N	Y	N	N		
4. Were controls selected or recruited from the same or similar population that gave rise to the cases (including the same timeframe)?	Y	Y	Y	Y	Y	Y		
5. Were the definitions, inclusion and exclusion criteria, algorithms or processes used to identify or select cases and controls valid, reliable, and implemented consistently across all study participants?	Y	Y	Y	Y	Y	Y		
6. Were the cases clearly defined and differentiated from controls?	Y	Y	Y	Y	Y	Y		
7. If less than 100 percent of eligible cases and/or controls were selected for the study, were the cases and/or controls randomly selected from those eligible?	N	N	N	N	N	N		
8. Was there use of concurrent controls?	NR	NR	NR	NR	NR	NR		
9. Were the investigators able to confirm that the exposure/risk occurred prior to the development of the condition or event that defined a participant as a case?	Y	Y	Y	Y	Y	Y		
10. Were the measures of exposure/risk clearly defined, valid, reliable, and implemented consistently (including the same time period) across all study participants?	Y	Y	Y	Y	Y	Y		
11. Were the assessors of exposure/risk blinded to the case or control status of participants?	N	N	N	N	N	N		
12. Were key potential confounding variables measured and adjusted statistically in the analyses? If matching was used, did the investigators account for matching during study analysis?	N	N	N	N	N	N		
Quality of the cohort study (score)	7	7	7	8	7	7		

Y= Yes, N= No; The maximum possible score on this scale is 12. “Good” was defined as a total score of 8-12; “fair” as a score 5-7, and “poor” as a score of less than 5.

### Statistical analysis

A meta-analysis of proportions was conducted using the Freeman-Tukey analysis under random-effects model to determine pooled estimates with a 95% confidence interval (CI). A random-effects model was used for differences among studies such as age, sex, surgical approaches, body mass index, indications for THA procedure, implant brand and methodology. A standard multivariate linear regression analysis (β) was performed to determine potential factors for implant failure or improved functional outcome. We completed all analyses with the Comprehensive Meta-Analysis (CMA) software, version 3 (Biostat, Englewood, New Jersey, USA) and significance was defined as p < 0.05.

## Results

We identified 1123 studies according to our search strategy. We removed 714 duplicate records and 232 studies after reading the title and abstract. Another 58 studies were excluded after reading the full text as the studies did not meet the inclusion criteria: studies on different outcome domains (n=21), mixed etiologies (n=12), 1st generation DM designs (n=10), cemented liner to cup (n=9), cadaveric or in vitro studies (n=3), studies not written in English (n=3). After exclusion, a total of 119 studies were included [41–115, 120–163] (Figure 1). Of these studies, 45 were case-control studies while 74 were case series. Since the objectives of this study were to validate the risk factors and modes of failures in the modern dual mobility implants, we extracted only the dual mobility group but not the control group from the 45 case-control studies.

### Baseline characteristics

This study included 30016 patients who had undergone DM-THA for primary and revision THA procedures. The mean age was 71.9 years (range, 19.2 to 87.6) and 63.2% of the patients were female. Mean follow-up duration in overall, primary, revision and recurrent dislocation group were 47.29 months (range, 3 to 152.4), 40.86 months (range, 3 to 152.4), 61.82 months (range, 6 to 87.6), and 35.23 months (range, 24 to 55), respectively. DM-THA was used in 19819 primary THA procedures, 9411 revision THA procedures and 786 revision THA procedures for recurrent dislocation.

#### Aseptic loosening

A total of 105 studies, including 28980 DM-THA procedures, reported the rate of aseptic loosening. The pooled rate was 1.6% (95% CI 0.008 – 0.032). The aseptic loosening rate in primary THA, revision THA and revision THA for recurrent dislocation were 0.9%, 2.2% and 2.4%, respectively (Table 4, Figure S1). A multivariate regression analysis revealed that a revision THA procedure for all causes (β=1.30, 95% CI 0.71 – 1.89), or for recurrent dislocation (β=1.18, 95% CI 0.26 – 2.10), carried a higher risk of aseptic loosening compared with a primary THA procedure (Table 5).

**Table 4:** Pooled event rate and clinical performance stratified by indications

	Rate or Mean Value	95% CI
Aseptic loosening		
Primary THA	0.009	0.007-0.012
Revision THA	0.022	0.016-0.030
Recurrent dislocation	0.024	0.013-0.045
Overall	0.016	0.008-0.032
Septic loosening		
Primary THA	0.008	0.006-0.011
Revision THA	0.023	0.017-0.032
Recurrent dislocation	0.025	0.013-0.049
Overall	0.016	0.007-0.037
Extra-articular dislocation		
Primary THA	0.006	0.005-0.008
Revision THA	0.013	0.009-0.017
Recurrent dislocation	0.025	0.014-0.043
Overall	0.012	0.006-0.025
Intra-prosthetic dislocation		
Primary THA	0.008	0.006-0.010
Revision THA	0.010	0.007-0.015
Recurrent dislocation	0.016	0.008-0.031
Overall	0.010	0.007-0.015
Periprosthetic fracture		
Primary THA	0.009	0.007-0.011
Revision THA	0.009	0.006-0.012
Recurrent dislocation	0.013	0.006-0.025
Overall	0.009	0.008-0.011
Implant failure		
Primary THA	0.023	0.018-0.030
Revision THA	0.055	0.042-0.073
Recurrent dislocation	0.060	0.034-0.103
Overall	0.042	0.021-0.081
Harris Hip score		
Primary THA	89.47	87.62-91.33
Revision THA	81.89	78.96-84.83
Recurrent dislocation	82.65	77.41-87.89
Overall	84.87	78.99-90.76
Merle d’Aubign*é* score		
Primary THA	17.08	16.85-17.30
Revision THA	15.45	15.07-15.83
Recurrent dislocation	16.57	15.85-17.28
Overall	16.36	15.20-17.53

THA: total hip arthroplasty.

**Table 5:** Multivariate linear regression analysis

Independent Variable	*β*-Coefficient	95% Confidence Interval	P Value
Aseptic loosening			
Age	-0.02	-0.05 – 0.01	0.269
Female Sex	0.55	-2.08– 3.17	0.683
Posterolateral approach (ref to others)	0.18	-0.59 – 0.94	0.654
BMI	-0.07	-0.19 – 0.06	0.302
Indication (ref to primary THA)			
Revision THA	1.30	0.71 – 1.89	<0.001
Recurrent dislocation	1.18	0.26 – 2.10	0.012
Septic loosening			
Age	-0.02	-0.05 – 0.01	0.226
Female Sex	1.39	-1.54 – 4.32	0.353
Posterolateral approach (ref to others)	0.34	-0.42 – 1.10	0.384
BMI	-0.09	-0.20 – 0.02	0.125
Indication (ref to primary THA)			
Revision THA	1.85	1.26– 2.44	<0.001
Recurrent dislocation	1.40	0.45 – 2.36	0.004
Extra-articular dislocation			
Age	0.01	-0.03 – 0.05	0.741
Female Sex	1.18	-1.82 – 4.18	0.440
Posterolateral approach (ref to others)	-0.39	-1.20 – 0.41	0.338
BMI	-0.10	-0.24 – 0.03	0.126
Indication (ref to primary THA)			
Revision THA	1.02	0.30 – 1.73	0.006
Recurrent dislocation	0.78	-0.49 – 2.04	0.230
Intra-prosthetic dislocation			
Age	0.00	-0.05 – 0.04	0.829
Female Sex	1.30	-2.04 – 4.64	0.444
Posterolateral approach (ref to others)	-0.31	-1.19 – 0.56	0.482
BMI	-0.05	-0.18 – 0.08	0.473
Indication (ref to primary THA)			
Revision THA	0.52	-0.24 – 1.28	0.180
Recurrent dislocation	0.88	-0.19 – 1.94	0.107
Periprosthetic fracture			
Age	-0.02	-0.06– 0.02	0.340
Female Sex	0.81	-2.47 – 4.08	0.629
Posterolateral approach (ref to others)	0.21	-0.70 – 1.12	0.651
BMI	-0.07	-0.22 – 0.08	0.364
Indication (ref to primary THA)			
Revision THA	0.93	0.23 – 1.62	0.009
Recurrent dislocation	0.42	-0.93 – 1.77	0.542
Implant failure			
Age	-0.04	-0.07 – -0.02	0.002
Female Sex	3.34	0.91 – 5.78	0.007
Posterolateral approach (ref to others)	0.34	-0.32 – 1.01	0.309
BMI	-0.06	-0.16 – 0.05	0.273
Indication (ref to primary THA)			
Revision THA	1.48	0.93 – 2.03	<0.001
Recurrent dislocation	1.08	0.24 – 1.92	0.012
Harris Hip score			
Age	-0.01	-0.34 – 0.32	0.964
Female Sex	3.66	-15.82 – 23.15	0.713
Posterolateral approach (ref to others)	-1.71	-8.11 – 4.69	0.601
BMI	0.58	-0.48 – 1.64	0.285
Indication (ref to primary THA)			
Revision THA	-9.44	-15.17 – -3.72	0.001
Recurrent dislocation	-6.81	-15.42 – 1.80	0.121
Merle d’Aubign*é* score			
Age	0.03	-0.03 – 0.09	0.378
Female Sex	-4.10	-8.17 – -0.03	0.049
Posterolateral approach (ref to others)	0. 23	-0.64 – 1.11	0.600
BMI	0.14	-0.03 – 0.31	0.109
Indication (ref to primary THA)			
Revision THA	-0.38	-1.45 – 0.69	0.487
Recurrent dislocation	-0.37	-1.81 – 1.07	0.617

BMI: body mass index; ref: reference; THA: total hip arthroplasty

#### Septic loosening

A total of 105 studies, including 28980 DM-THA procedures, reported septic loosening rates. The pooled rate was 1.6% (95% CI 0.007 – 0.037). The septic loosening rate in primary THA, revision THA and revision THA procedure for recurrent dislocation were 0.8%, 2.3% and 2.5%, respectively (Table 4, Figure S2). A multivariate regression analysis showed that both revision THA for all causes (β=1.85, 95% CI 1.26 – 2.44) and for recurrent dislocation (β=1.40, 95% CI 0.45 – 2.36) were at a higher risk of septic loosening, compared with a primary THA procedure (Table 5).

#### Extra-articular dislocation

A total of 113 studies, including 20447 DM-THA procedures, presented the extra-articular dislocation rate. The pooled rate was 1.2% (95% CI 0.006 – 0.025). The extra-articular dislocation rate in primary THA, revision THA and revision THA for recurrent dislocation were 0.6%, 1.3% and 2.5%, respectively (Table 4, Figure S3). Compared with a primary THA procedure, risk of dislocation was higher after revision THA procedures (β=1.02, 95% CI 0.30 – 1.73) (Table 5).

#### Intra-prosthetic dislocation

A total of 113 studies, including 20447 DM-THA procedures, reported the intra-prosthetic dislocation rate. The overall rate was 1.0% (95% CI 0.007 – 0.015). The intra-prosthetic dislocation rate in primary THA, revision THA and revision THA for recurrent dislocation were 0.8%, 1.0% and 1.6%, respectively (Table 4, Figure S4). None of the factors including age, female sex, posterolateral approach, BMI or indication have led to intra-prosthetic dislocation (Table 5).

#### Periprosthetic fracture

A total of 100 studies, including 27731 DM-THA procedures, recorded the periprosthetic fracture rate. The pooled rate was 0.9% (95% CI 0.008 – 0.011). The periprosthetic fracture rates in primary THA, revision THA and revision THA for recurrent dislocation were 0.9%, 0.9% and 1.3%, respectively (Table 4, Figure S5). Revision THA procedure for all causes (β=0.93, 95% CI 0.23 – 1.62) was a risk factor for periprosthetic fracture (Table 5).

#### Overall implant failure

A total of 105 studies, including 27873 DM-THA procedures, recorded the implant failure rate. The pooled rate was 4.2% (95% CI 0.021 – 0.081) at a mean follow-up of 45.8 months. The implant failure rates in primary THA, revision THA and revision THA for recurrent dislocation were 2.3%, 5.5% and 6.0%, respectively (Table 4, Figure S6). Younger age (β=-0.04, 95% CI -0.07 – -0.02), female sex (β=3.34, 95% CI 0.91 – 5.78), revision THA procedure for all causes (β=1.48, 95% CI 0.93 – 2.03) and for recurrent dislocation (β=1.08, 95% CI 0.24 – 1.92) were risk factors for implant failures (Table 5).

#### Functional outcome

We included 49 (N= 7086) and 21 (N= 2764) studies that evaluated the functional outcome using Harris hip score and Merle d’Aubign*é* score. The pooled Harris hip score and Merle d’Aubign*é* score were 84.87 (95% CI 78.99 – 90.76) and 16.36 (95% CI 15.20 – 17.53), respectively (Table 4, Figure S7, S8). Revision THA procedure for all causes (β=-9.44, 95% CI -15.17 – -3.72) and female sex (β=-4.10, 95% CI -8.17 – -0.03) were associated with lower functional scores. (Table 5).

## Discussion

In this meta-analysis, we included 119 studies with 30016 primary and revision THA procedures using the modern DM design. At a mean follow-up of 47.3 months, the overall failure rate of modern dual mobility design was 4.2%. The most common failure modes include aseptic loosening (primary: 0.9%, revision for all causes: 2.2%, revision for recurrent dislocation: 2.4%), septic loosening (primary: 0.8%, revision for all causes: 2.3%, revision for recurrent dislocation: 2.5%), extra-articular dislocation (primary: 0.6%, revision for all causes: 1.3%, revision for recurrent dislocation: 2.5%), intra-prosthetic dislocation (primary: 0.8%, revision for all causes: 1.0%, revision for recurrent dislocation: 1.6%) and periprosthetic fracture (primary: 0.9%, revision for all causes: 0.9%, revision for recurrent dislocation: 1.3%). The multi-regression analysis revealed that revision THA procedures were associated with a higher risk of aseptic loosening, septic loosening, extra-articular dislocation, periprosthetic fracture, overall implant failure and lower Harris Hip scores. Interestingly, several risk factors that were identified for THA dislocation such as advanced age, female sex, posterolateral approach and increased BMI were not risk factors for extra-articular dislocation. On the other hand, younger and female patients were associated with higher risk of implant failure. In terms of functional outcome, the patients were satisfied with their postoperative function based on the improved Harris hip score and Merle d’Aubigné score.

Dislocation is one of the common causes of THA implant failure and can be caused by many factors [8]. In current literature, the known risk factors include advanced age, female patients [9, 10], obesity [11, 12], previous hip surgeries [13], posterolateral surgical approach [14, 15], THA for acute fractures, patients with neurological diseases [16], and patients with abductor weakness [17, 18]. The dual mobility design increases femoral head-to-neck ratio and jump distance to improve stability [20–23]. Therefore, we can anticipate decreased dislocation rates for the DM design in primary and revision THA. Even after revision THA due to recurrent instability, the dislocation rate was only 2.5%, which was much lower than the reported dislocation rate after primary THAs and revision THAs, which ranged from 0.3% to 10% [2–4] and 5% to 30% [5–7], respectively. In addition, a multivariate analysis revealed that older age, female patients, posterolateral approach and BMI were not risk factors for dislocation after DM-THA. Based on the difference in risk factors for dislocations, we can assume that the DM design can effectively overcome some of the shortcomings of previous THA designs. Nevertheless, optimization of component position and restoration of soft tissue tension are paramount to prevent dislocation in both primary and revision THA procedures.

Despite these improvements, there are still some concerns with the DM design, including increased wear of the acetabular liner [164], increased risk of aseptic loosening [30] and intra-prosthetic dislocation [30].

The two-articulation design creates two surfaces for plastic deformation and wear, which theoretically leads to a higher wear rate than fixed-bearing THA. The inner, small articulation dominates the majority of movement and follows the Charnley’s low-friction principle with a small-diameter head to reduce wear [20]. The motion between the outer shell and acetabular component occurs in extreme angle when femoral neck abuts the PE liner and creates a homogenous wear over the liner [40]. Using plain radiographs or implant retrieval analysis, several studies aimed to assess the volumetric difference in wearing of DM articulations and fixed-bearing THA [165–172]. Interestingly, the wear rate of ultra-high molecular weight polyethylene (UHMWPE) bearing in the 1^st^ generation DM cup was less than 40 mm^3^/year, which was similar to wear rate of UHMWPE in fixed-bearing THAs (30–80 mm^3^/year at 15 to 21 year follow up) [165–169]. In vitro simulation study for modern generation DM cup, using highly cross-linked polyethylene (HXLPE), reported lower wear rate in DM cup compared to fixed-bearing THA (1.2 vs. 2.7 mm^3^/million cycles, respectively) [170]. In another study performed by Laende et al., the wear rate of modern generation DM cups with HXLPE at 3 years follow-up was 0.02 mm/year in DM cup, which was similar to non-dual mobility constructs (0.00 to 0.06 mm/year) [69, 171]. In contrast, Deckard et al. recorded the wear rate was two times higher for modern-generation DM cup with HXLPE than the fixed-bearing THA (0.27mm/year and 0.11 mm/year, respectively) [172]. The in vitro simulation or retrieval studies have validated reasonable wear rates of DM articulation using either UHMWPE or HXLPE [165–170]. The results from studies using plain radiographs to estimate the wear rate were controversial, which is considered less accurate than the retrieval or simulation studies [171, 172]. Currently, there is limited evidence regarding the increased PE wear of modern DM articulation.

The non-porous alumina-coated surface, tripod anchoring system of acetabular component and polyethylene wear have been associated with a higher aseptic loosening rate in the first-generation DM implants [24, 29, 31]. Several changes have been made in modern dual mobility designs, including [1] to replace UHMWPE with HXLPE to reduce wear [33, 34]; (2) to add bevelled edges (or chamfer) in polyethylene (PE) inserts to lower femoral neck impingement and wear [32]; (3) press-fit fixation by bilayer coating of porous titanium and hydroxyapatite to enhance osseointegration on the outer surface [31]; (4) modular metal liner design to facilitate supplementary screw fixation. The long-term overall survival and aseptic loosening rate of the primary THAs using 1^st^ generation DM implants were 85-95.4% and 3-8.3%, respectively [24–28]. In this study, the primary THAs using modern generations DM implants are associated with a better overall survival (97.7%) and a lower aseptic loosening rate (0.9%). This pooled aseptic loosening rate was comparable to that of primary, fixed-bearing THA from several registries, which ranged from 0.7-1.1% at 5 to 16 years [1, 173, 174].

The modern, modular design has an additional cobalt-chromium (CoCr) liner inserted into a titanium acetabular component allowing supplementary screw fixation to enhance primary stability. However, the metal-on-metal interface between CoCr liner and titanium cup is at risk of fretting corrosion and remains a concern [175–177]. Metal ions can further lead to advance local tissue reaction (ALRT) and implant loosening [178]. The first study regarding metal ions was conducted by Matsen Ko et al., which revealed 21% of the patient had elevated serum chromium levels [179]. Other studies reported that serum ion levels (cobalt, chromium or titanium) was elevated in 9.3-23% of the patients [47, 111]. On the other hand, some studies have noted that this elevation was not associated with clinical adverse events including instability, loosening or need of revision [64, 67, 72]. In summary, the current evidence suggests there is a slight elevation of serum ion level but this does not negatively affect the implant survival.

Intra-prosthetic dislocation (IPD) is a rare complication of DM design, which occurs as a result of retentive failure of the inner articulation. Long-term, homogenous PE wear or impingement at extreme range of motion between neck and PE liner leads to loss of PE retentive rim and IPD [180, 181]. The incidence of IPD ranged from 0.7%-4.3% in first generation of DM cup and [29, 30] modifications have been made to the 2^nd^ generation DM implants. These changes include a thinner, more polished femoral neck to reduce impingement with the liner and the use of HXLPE to reduce wear during contact [32]. In this study, we noted a lower IPD rate with the modern design in primary THA and revision THA was 0.8% and 1.0% respectively, which is much lower than the 1^st^ generation [29, 30]. Another form of IPD has been observed in modern generation DM implants, which often occurs in the short-term. This form of IPD results from a secondary decapsulation of the liner followed by reduction for dislocation [182]. During close reduction of a dislocated DM-THA, impingement occurs between the PE liner and the posterior edge of the acetabular component. The excessive loading during reduction maneuver may “decapsulate” the femoral head from PE liner. Therefore, the reduction should be performed gradually under general anesthesia to reduce excessive muscle tension [29].

Our meta-analysis showed that the mid-term revision rates in primary and revision DM-THA were 2.3% and 5.5-6.0%, respectively. These results were comparable to the reported outcome of primary or revision, fixed-bearing THA [1, 38, 39, 60, 73, 98, 108, 183, 184]. In primary fixed-bearing THA, the mid-term and long-term revision rate ranged from 1.2-4.0% and 12.1-14.3%, respectively [1, 38, 60, 73, 98, 108, 183]. In revision fixed-bearing THA, the mid-term and long-term revision rates can be up to 5.3-13% and 27-45%, respectively [39, 184].

This meta-analysis revealed promising mid-term outcomes and a reduction in dislocation rate, but the long-term implant survival of modern DM-THA is still lacking. For revision THA procedures, younger age and female patients were associated with a higher risk of implant failure. Younger patients have been established as a risk factor for failure after primary THAs. However, whether female sex is a risk factor remains controversial [185–188]. This can be attributed to the representativeness of the study cohort, follow-up duration and type of implant. Although female patients have been associated with increased risk of dislocation, aseptic loosening, periprosthetic fracture and overall implant failure after primary THA [187, 188], the same was not seen in DM-THA aside from overall implant failure. Potential confounders and inadequate follow-up duration are important considerations when interpreting this result.

We should recognize several limitations. First, we only included studies which the full text was available in English. In addition, due to the nature of our research question, the level of evidence of the included studies was low (III or IV). Second, we included studies that reported outcome of modern DM (the 2^nd^ and 3^rd^ generation) implants over a time span of 12 years between 2008 to 2020. Modern DM-THA implants were developed in the 1990s, and the studies about modern DM-THA implants were mostly conducted after 2000. We could only analyze factors that were clearly described in the studies, including age, sex, surgical approach, BMI and indication for hip arthroplasty. Factors such as surgeons’ experience, patient activity level or implant designs could have affected the outcome but were unavailable and thus were not analyzed. Therefore, we considered articles that were conducted after 2000. Third, the protocol of this meta-analysis has not been registered, which can have a risk for reporting bias. Fourth, we did not include grey literature or unpublished studies in this work. Nonetheless, this review provides an updated review regarding the outcome of modern DM implants and factors that might affect the outcome.

## Conclusions

In conclusion, the mid-term implant survival of modern dual-mobility design was satisfactory. Aseptic loosening continues to be the most common failure mode after DM-THA. Younger age and female sex were correlated with implant failure.

## Supplementary Information

**Additional file 1:Figure S1.** Forest plot of the pooled aseptic loosening rate among included studies.

**Additional file 2: Figure S2.** Forest plot of the pooled septic loosening rate among included studies.

**Additional file 3: Figure S3.** Forest plot of the pooled extra-articular dislocation rate among included studies.

**Additional file 4: Figure S4.** Forest plot of the pooled intra-prosthetic dislocation rate among included studies.

**Additional file 5: Figure S5.** Forest plot of the pooled periprosthetic fracture rate among included studies.

**Additional file 6: Figure S6.** Forest plot of the pooled implant failure rate among included studies.

**Additional file 7: Figure S7.** Forest plot of the pooled Harris hip score among included studies.

**Additional file 8: Figure S8.** Forest plot of the pooled Merle d’Aubigné score among included studies.

## Data Availability

As this is a review and meta-analysis, we completed a comprehensive search on PubMed, MEDLINE, Cochrane Reviews and Embase for studies. All data generated or analysed during this study are included in this published article [and its supplementary information files]

## Abbreviations

DM: Dual mobility
THA: total hip arthroplasty
BMI: body mass index
IPD: intra-prosthetic dislocation
PRISMA: Preferred Reporting Items for Systematic Reviews and Meta-analysis
CI: confidence interval
CMA: Comprehensive Meta-Analysis
UHMWPE: ultra-high molecular weight polyethylene
HXLPE: highly cross-linked polyethylene
PE: polyethylene
CoCr: cobalt-chromium
ALRT: advance local tissue reaction
