# Supplementary figures and images for: Risk factors and modes of failure in the modern dual mobility implant. A systematic review and meta-analysis
Source: BMC Musculoskelet Disord. 2021 Jun 14;22:541. doi: 10.1186/s12891-021-04404-4 (PMC8204435; doi:10.1186/s12891-021-04404-4)

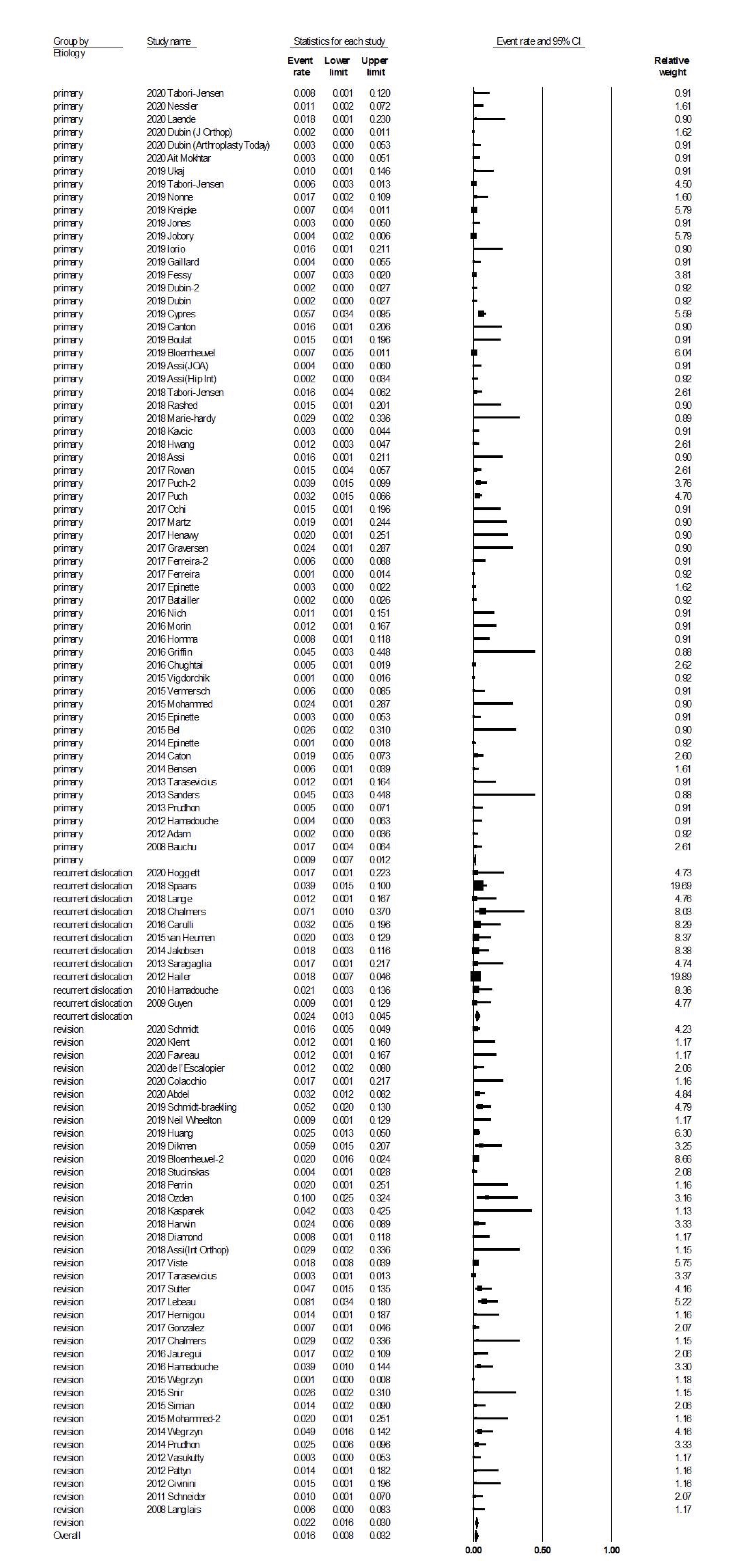

Supplement: Supplementary file 1 — Additional file 1:Figure S1. Forest plot of the pooled aseptic loosening rate among included studies. [file 12891_2021_4404_MOESM1_ESM.jpg]

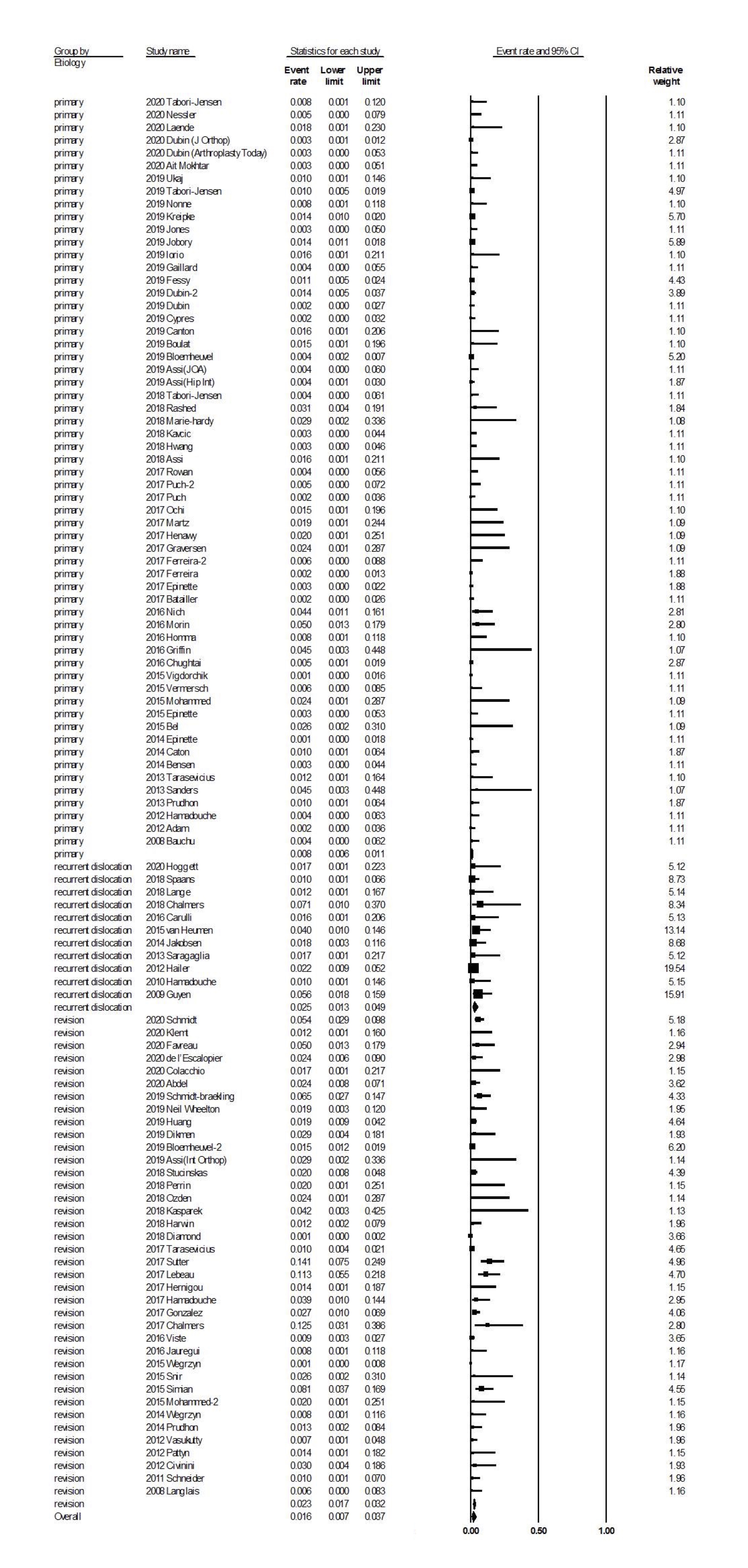

Supplement: Supplementary file 2 — Additional file 2: Figure S2. Forest plot of the pooled septic loosening rate among included studies. [file 12891_2021_4404_MOESM2_ESM.jpg]

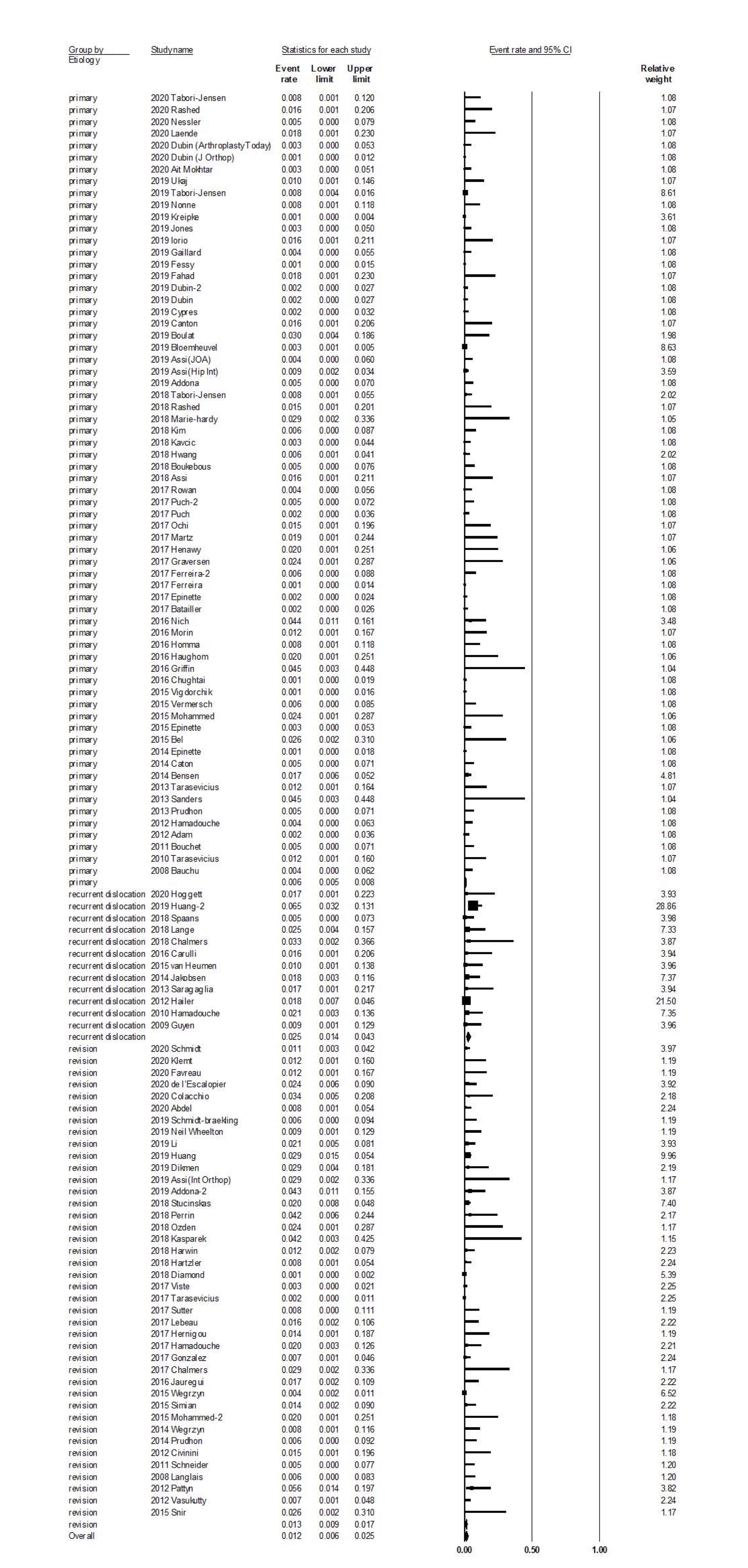

Supplement: Supplementary file 3 — Additional file 3: Figure S3. Forest plot of the pooled extra-articular dislocation rate among included studies. [file 12891_2021_4404_MOESM3_ESM.jpg]

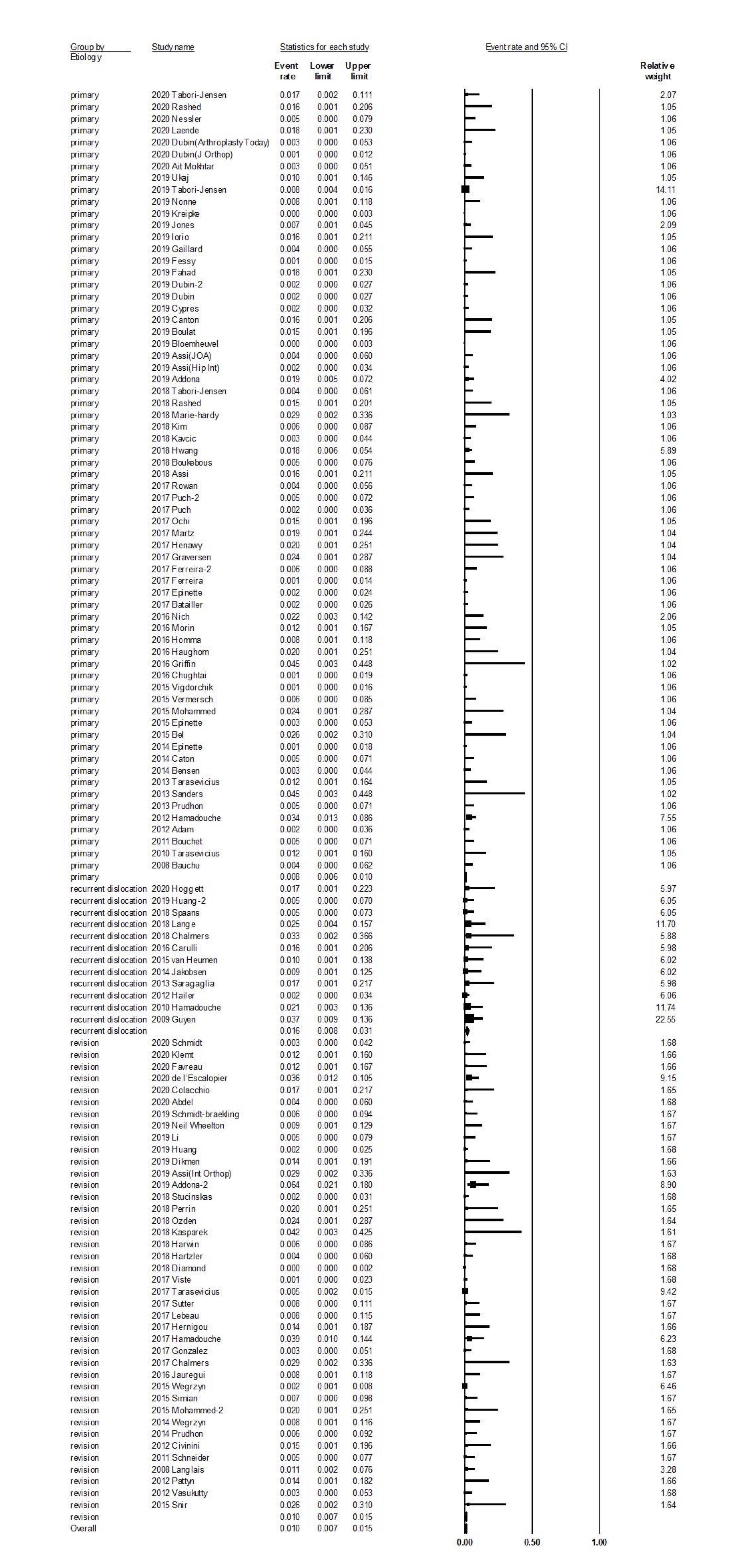

Supplement: Supplementary file 4 — Additional file 4: Figure S4. Forest plot of the pooled intra-prosthetic dislocation rate among included studies. [file 12891_2021_4404_MOESM4_ESM.jpg]

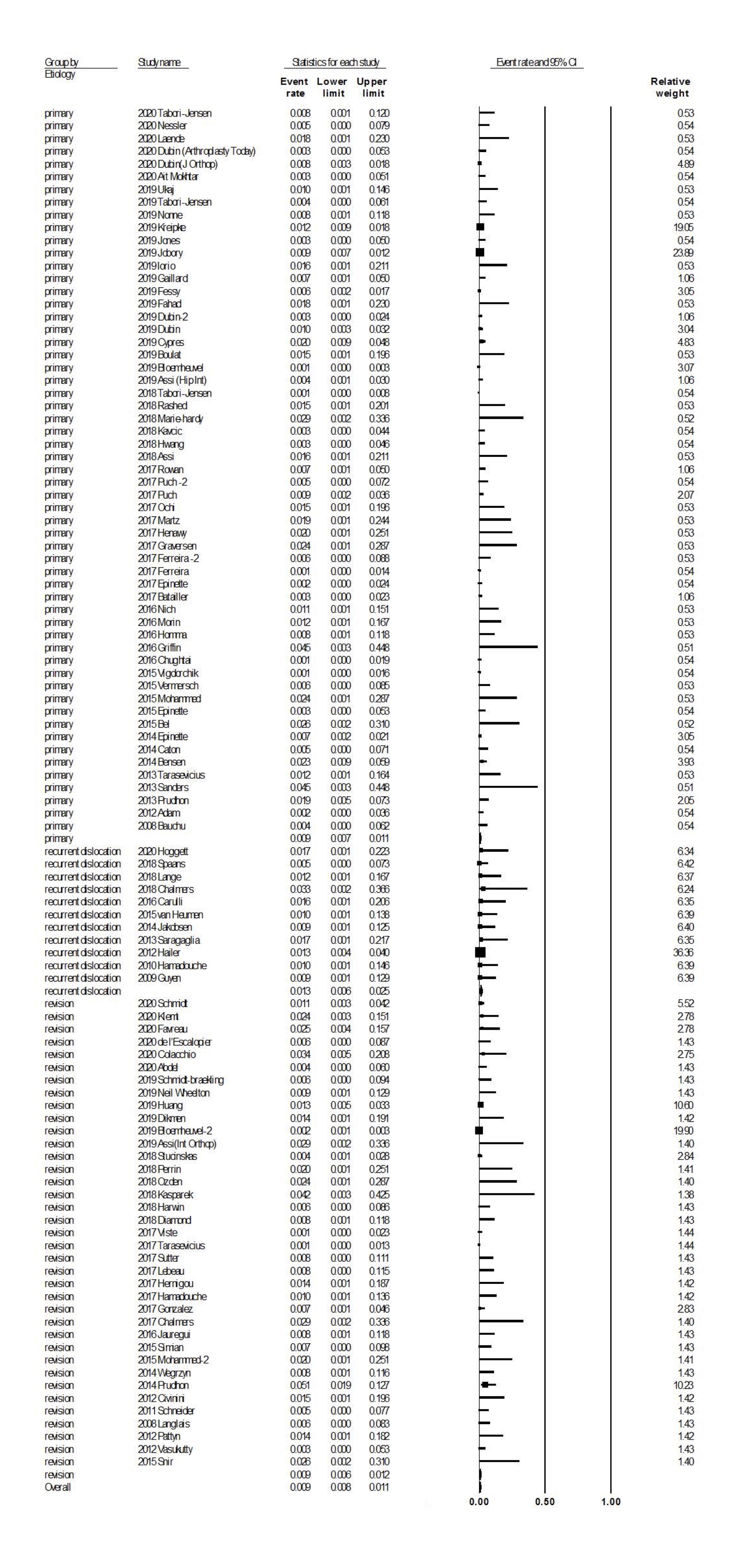

Supplement: Supplementary file 5 — Additional file 5: Figure S5. Forest plot of the pooled periprosthetic fracture rate among included studies. [file 12891_2021_4404_MOESM5_ESM.jpg]

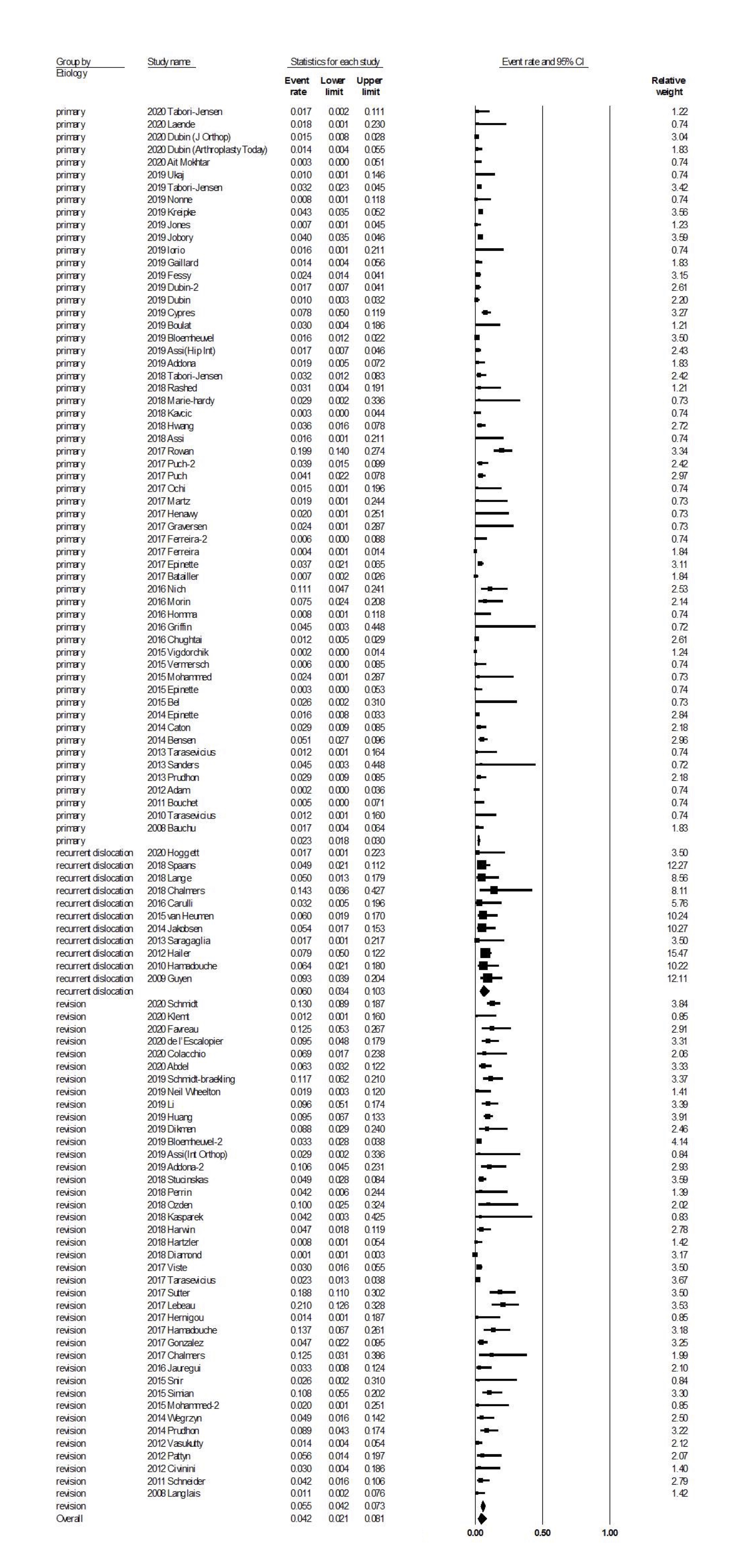

Supplement: Supplementary file 6 — Additional file 6: Figure S6. Forest plot of the pooled implant failure rate among included studies. [file 12891_2021_4404_MOESM6_ESM.jpg]

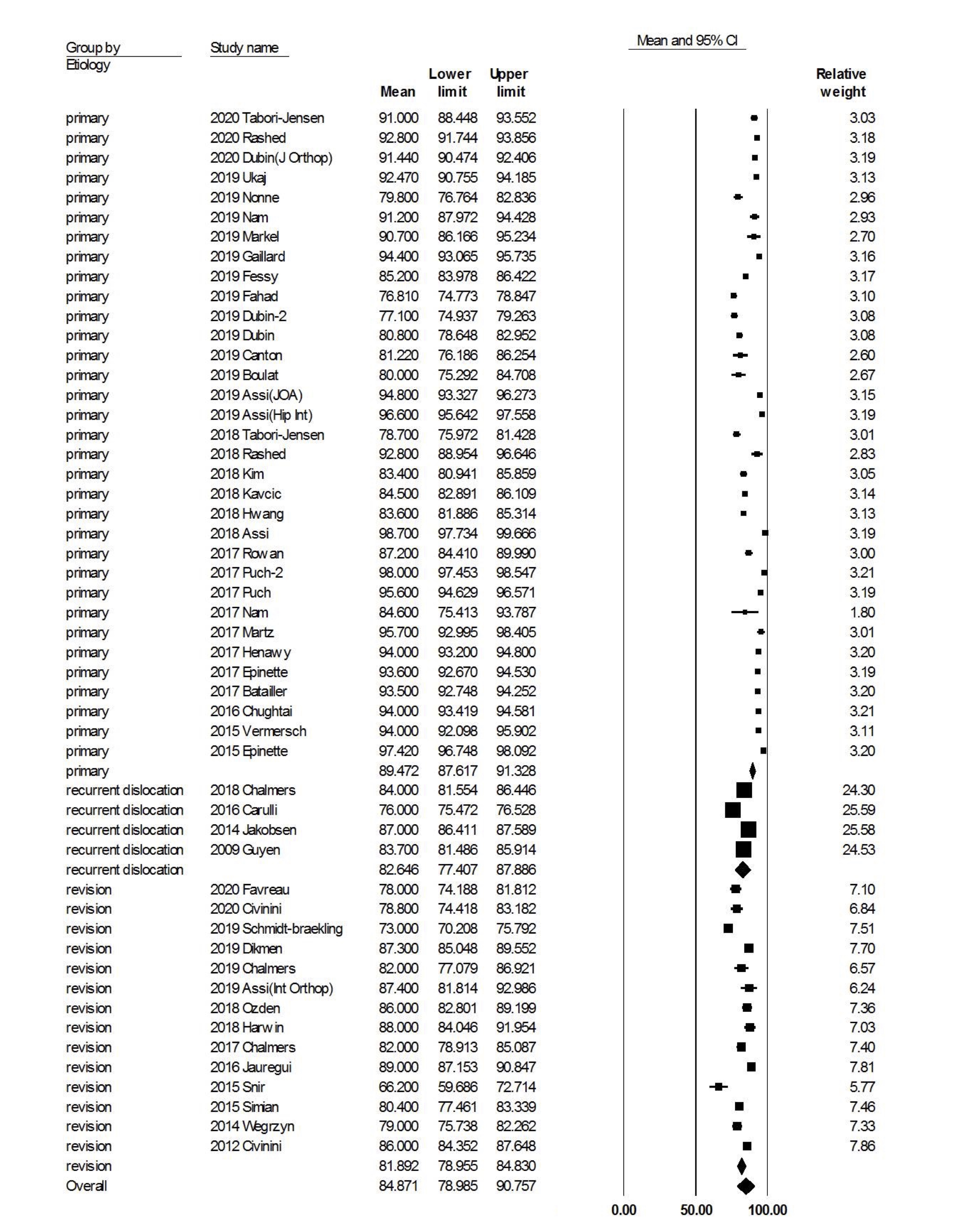

Supplement: Supplementary file 7 — Additional file 7: Figure S7. Forest plot of the pooled Harris hip score among included studies. [file 12891_2021_4404_MOESM7_ESM.jpg]

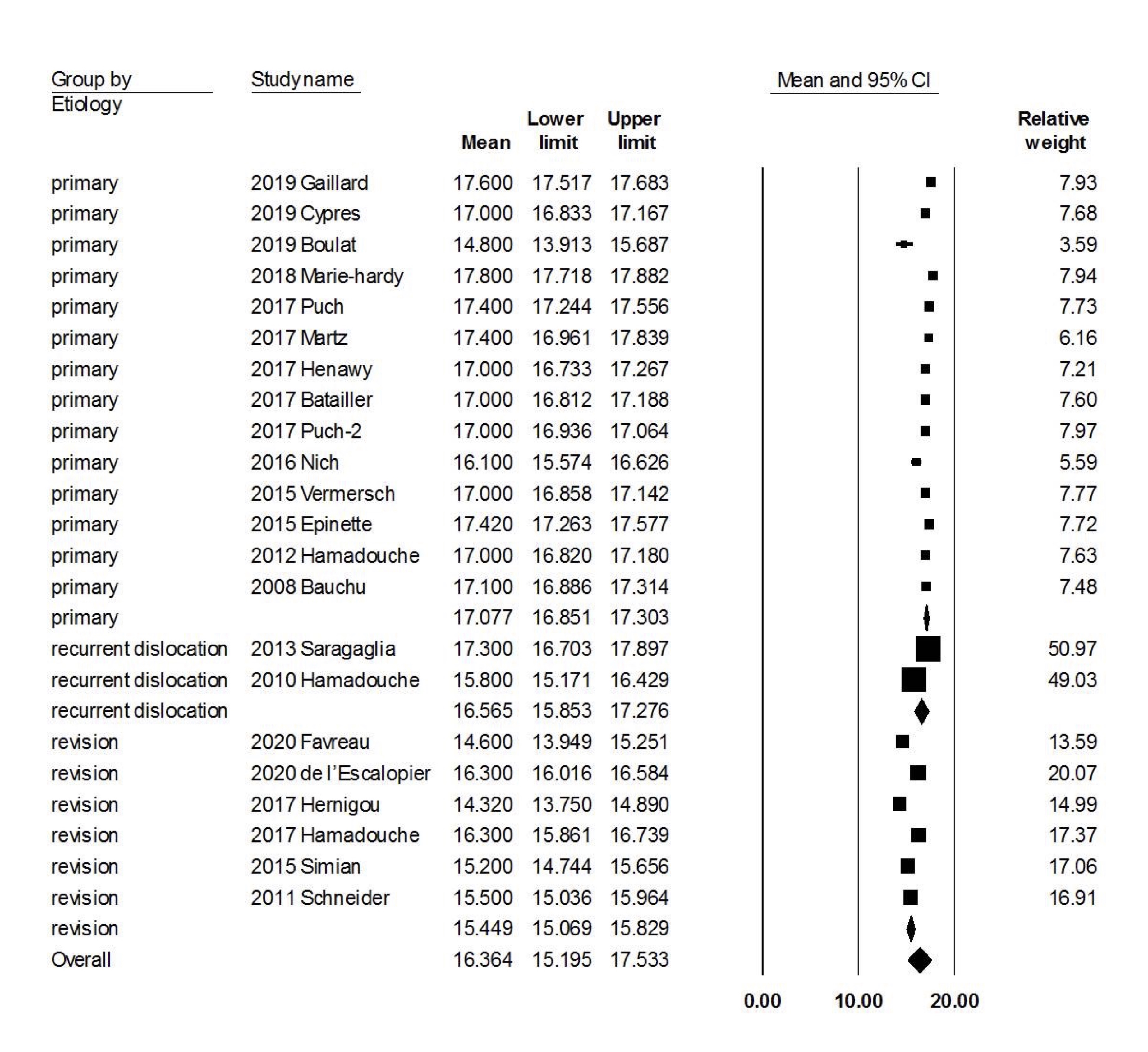

Supplement: Supplementary file 8 — Additional file 8: Figure S8. Forest plot of the pooled Merle d’Aubigné score among included studies. [file 12891_2021_4404_MOESM8_ESM.jpg]
